# Graduations in the University of Edinburgh

**Published:** 1837-10

**Authors:** 


### GRADUATIONS IN THE UNIVERSITY OF EDINBURGH.

The following is a list of the gentlemen, 105 in number, who received the degree of Doctor of Medicine at the University of Edinburgh, Tuesday, August 1; with the titles of their theses:

H. C. Barlow, on the causes and effects of disease, considered in reference to the moral constitution of man.
T. H. Burgess, on paralysis.
William Brown, on morphology.
W. H. Brownson, on the nature and treatment of gunshot wounds, and their consequences.
William Bayard, on Asiatic cholera.
Robert Butler, on apoplexy.
J. H. Bennett, on the physiology and pathology of the brain.
J. H. Branfoot, on the acquired perceptions of hearing.
J. O'B. M. Barry, on endocarditis.
James Barlas, on artificial pupil.
P. J. Barry, on rheumatism.
J. E. Cummins, on ophthalmia.
G. S. Carden, on temporary insanity.
George Cossar, on inguinal hernia.
Joseph Cartmell, on pseudo-pathological appearances.
J. R. Cormack, on air in the organs of circulation.
Francis Cooke, on pellagra.
T. W. Curtis, on the functions of the cæcum.
Michael Cormack, on asthma.
C. Chadwick, How far are secretion and nutrition dependent on nervous influence?
J. R. H. Couson, on the nature, symptoms, and treatment of bronchitis.
A. W. Campbell, on acute pericarditis, particularly as connected with rheumatism.
Thomas Crawford, on paralysis.
Alexander Duncan, on hernia.
R. H. Davidson, on the organs and physiology of digestion.
Hugo Donaldson, on bronchitis.
S. P. C. Evans, on equivocal, comparative, and human generation.

James Edwards, on neuralgia.

John Fortune, on acute hepatitis.

J. W. Fullerton, on mortification.

George Frazer, on the connexion of nervous energy with muscular contractility.

Thomas Gordon, on erysipelas.

F. W. Grant, on the nature, diagnosis, and treatment of aneurism; with a few remarks on the spontaneous suppression of hemorrhage.

John Grant, What are the relative advantages of different trades and professions, as regards their compatibility with bodily health?

Alexander Greig, on asphyxia.

W. T. Geary, on the hydrated peroxide of iron as an antidote to arsenic.

G. D. Gordon, on acute dysentery.

John Houseman, on the morbid affections of old age.

A. Hunter, on the pathology and treatment of granular disease of the kidney.

S. Hunter, a practical treatise on ruptured urethra, produced by external violence.

George Hood, on aneurism and its treatment.

William Hilliard, some general considerations on phlebitis.

Thomas Hayle, De necrosi.

William Hey Hodgson, on apoplexy.

E. Johnson, on the anatomy of the mammary gland.

J. Johnston, on the tongue, pulse, and urine, as indications of health and disease.

James Jopp, on angina pectoris.

H. Kinglake, on the physiology of digestion.

G. Kennion, a sketch of some of the principal diseases which are peculiar or incident to the puerperal state.

W. D. Kingdon, on scirrhus.

P. G. Kennedy, observations on the general pathology of diseases of the eye, with remarks on the characters which are diagnostic of the idiopathic and symptomatic ophthalmia.

Adam Lyszeznski, on small-pox.

S. D. Lees, on the pathology of the ear.

T. H. Lowry, on tetanus.

George Lund, on the use of the thyroid and thymus glands, and of the spleen and suprarenal capsules.

George Aaron Martin, on scorbutus.

W. H. Madden, on the connexion between the muscles and nervous system.

L. M'Lean, an account of the bilious remittent fever, more particularly as it occurs in the West Indies.

Alexander Ross Morton, on dysentery.

H. Montgomery, on the origin and mode of formation of tubercles in the lungs.

W. O. Mackenzie, on the distinctive characters, classes, and treatment of those ulcers (including hospital gangrene,) which have lately been prevalent in the British army and navy, on some foreign stations.

George Parker May, on croup.

James Mitchell, on scarlatina.

John Morison, on apoplexy.

J. L. Marsden, De kenothumia, or ennui.

H. R. Melville, on the effects of climate and food on man.

M. R. Mahony, some observations on aneurism and diseases of the heart, with case.

G. S. Newbigging, on the effusion and organization of coagulable lymph.

John O'Brien, on the hygiène of infants.

J. C. Orgile, on the morbid effects occasionally induced by the operation of mercury.

Eugene O'Neile, on vesicular emphysema.

James H. Pring, on chorea.

William Pringle, on phagedena gangrænosa.

J. P. Phipps, on cold affusion in croup.

Arthur Powell, on the causes of disease.

Charles Ronayne, on cancer of the uterus.

J. S. Reid, on variola.

W. A. Reeves, on delirium tremens.

E. C. Seaton, on the powers which move the blood.

William Scott, observations upon pseudo-inflammatory affections.

R. Skerrett, on malignant or Asiatic cholera.

M. Satterthwaite, on pathological chemistry.

George Smyth, on ascites.
J. P. Shuman, on the pathology of dropsy.
John Spowart, on tetanus.
William Stanger, on cynanche trachealis.
J. H. Shirreff, What advantage do we derive from auscultation in detecting pregnancy?
J. C. Sortain, on the function of the cæcum.
Thomas Stratton, on chronic rheumatism.
T. H. Shute, on fever; being an enquiry into its intimate nature and causes.
James Satchell, on the signs of pregnancy.
William Scott, on scarlatina.
Thomas R. Scott, on wounds of the thorax.
Henry Hunt Stubb, on influenza.
F. N. Slight, on injuries of the head from external violence.
H. H. Turnbull, on venous inflammation.
A. S. Thomson, observations on the influence of climate on the health and mortality of the inhabitants of the different regions of the globe.
George F. Thomson, on croup.
William Tatlock, on hydrocele.
B. W. Wright, on the jungle fever of India.
J. G. Wood, Under what circumstances is the operation of trephining to be had recourse to?
J. Waters, on hypertrophy of the heart, and on that of the left ventricle in particular.
J. W. Wallace, on hydrocele of the tunica vaginalis testis.
James Balfour Wishart, on abortion.

It has been an object lately with the Senatus to improve the character of the theses, and with this view it was advertised that a gold medal would be given for the best production of this class. This plan appears to have been eminently successful; a large number of most excellent theses having been given in. On the graduation day, Sir C. Bell, as dean of the Faculty, announced that Dr. W. H. Madden had obtained the medal for the best essay on Cutaneous Absorption. Dr. J. H. Bennett another, for the best Report of Surgical Cases. Dr. J. R. Cormack, and Dr. A. S. Thomson, one each for their respective theses. He stated that the latter two prizes had been awarded to those dissertations which contained the greatest amount of original information; but that the theses of the following gentlemen had likewise been selected by the professors as worthy of being so distinguished:—Dr. H. C. Barlow, Dr. J. H. Bennett, Dr. W. H. Madden, Dr. G. S. Newbigging, Dr. C. Chadwick, Dr. M. Satterthwaite, Dr. T. W. Curtis, Dr. — Hunter, Dr. E. C. Seaton, Dr. J. O'Brien, M. Barry.

We understand that the prize essays were all extremely good; and, as one of the conditions was to print, we anticipate that the profession generally will benefit by the mass of experiments and original observations which we understand to have been displayed in the respective dissertations. The medals, much to the disappointment of those present, were not forthcoming; a circumstance owing, no doubt, to the anxiety of the Senatus that the die about to be made for the occasion, and the medals themselves, should be worthy of the successful competitors, and the alma mater to which they belong.

---

